# Brain-derived proteins in the CSF, do they correlate with brain pathology in CJD?

**DOI:** 10.1186/1471-2377-6-35

**Published:** 2006-09-21

**Authors:** Constanze Boesenberg-Grosse, Walter J Schulz-Schaeffer, Monika Bodemer, Barbara Ciesielczyk, Bettina Meissner, Anna Krasnianski, Mario Bartl, Uta Heinemann, Daniela Varges, Sabina Eigenbrod, Hans A Kretzschmar, Alison Green, Inga Zerr

**Affiliations:** 1 National Reference Center for TSE Surveillance at the Dept. of Neurology, Georg-August-University Göttingen, Robert-Koch-Str. 40, 37075 Göttingen, Germany; 2 Dept. of Neuropathology, Georg-August-University Göttingen, Robert-Koch-Str. 40, 37075 Göttingen, Germany; 3 Institute of Neuropathology, LMU München, Feodor-Lynen-Str. 23, 81377 München, Germany; 4 National CJD Surveillance Unit, The University of Edinburgh, EH4 2XU Edinburgh, UK

## Abstract

**Background:**

Brain derived proteins such as 14-3-3, neuron-specific enolase (NSE), S 100b, tau, phosphorylated tau and Aβ_1–42 _were found to be altered in the cerebrospinal fluid (CSF) in Creutzfeldt-Jakob disease (CJD) patients. The pathogenic mechanisms leading to these abnormalities are not known, but a relation to rapid neuronal damage is assumed. No systematic analysis on brain-derived proteins in the CSF and neuropathological lesion profiles has been performed.

**Methods:**

CSF protein levels of brain-derived proteins and the degree of spongiform changes, neuronal loss and gliosis in various brain areas were analyzed in 57 CJD patients.

**Results:**

We observed three different patterns of CSF alteration associated with the degree of cortical and subcortical changes. NSE levels increased with lesion severity of subcortical areas. Tau and 14-3-3 levels increased with minor pathological changes, a negative correlation was observed with severity of cortical lesions. Levels of the physiological form of the prion protein (PrP^c^) and Aβ_1–42 _levels correlated negatively with cortical pathology, most clearly with temporal and occipital lesions.

**Conclusion:**

Our results indicate that the alteration of levels of brain-derived proteins in the CSF does not only reflect the degree of neuronal damage, but it is also modified by the localization on the brain pathology. Brain specific lesion patterns have to be considered when analyzing CSF neuronal proteins.

## Background

In recent years, the analysis of the cerebrospinal fluid (CSF) has become increasingly important to support the clinical diagnosis in patients with sporadic Creutzfeldt-Jakob disease (sCJD). Various brain-derived proteins have been studied in the CSF to date, such as 14-3-3 proteins, gamma-enolase (neuron-specific enolase, NSE), tau, phosphorylated tau, S-100b and Aβ_1–42 _[1-11]. These studies were mainly focused on diagnostic aspects. Elevated levels of these proteins were used as surrogate parameters for neuronal damage (14-3-3, tau, gamma-enolase) or astrocytic gliosis (S-100b), following the idea that CSF proteins reflect changes in pathological brain conditions.

Although a lot of information has been gained about abnormal CSF levels of these proteins, only few publications report on levels of particular proteins and disease stage or even brain pathology [12-16]. It was assumed that those protein levels correlate with neuronal damage or astrocytic gliosis. However, the question of whether particular protein level abnormalities reflect changes in certain brain areas affected has not been addressed so far.

In this study, we have investigated brain-derived proteins such as the physiological form of the prion protein (PrP^C^), 14-3-3 proteins, tau, phosphorylated tau, NSE and S-100b in cerebrospinal fluid (CSF) in patients with sCJD with respect to the neuropathological lesion profile in these patients.

## Methods

### Study design

#### Patients

The study population comprised 57 sporadic CJD according to criteria who were registered at the National Reference Center for TSE Surveillance in Göttingen from June 1993 to August 2003 with clinical data, CSF samples and brain lesion profile scoring available (see below) [17-19]. Iatrogenic and familial or genetic cases were excluded.

The study was approved by the Ethic Committee of the Medical Faculty in Goettingen, Germany, (approvals 11/11/93 from 18^th ^September 1996, amendments from 11/11/93 from 12^th ^September 2002 and 30/1/05 from 18^th ^February 2005). The informed consent of the relatives was obtained.

Clinical data concerning sex, date of birth/death, age at disease onset, disease duration, date of lumbar puncture, were collected from examination protocols and medical charts.

The analysis of the codon 129 genotype of the prion protein gene (*PRNP*) was performed after isolation of genomic DNA from blood according to standard methods [20]. None of the cases carried a mutation (n = 55). Because the primary goal of the study was to analyze the correlation between brain lesion profiles and CSF markers and because only of the limited numbers of PrP^Sc ^typing, data were not stratified by different molecular subtypes.

#### Neuropathology

Nine brain regions were investigated for the degree of spongiform changes, gliosis and nerve cell loss. They comprised five cortical regions (medial frontal gyrus, cingulate gyrus, inferior temporal gyrus, inferior parietal gyrus and area striata and parastriata), three subcortical regions (caudate nucleus, putamen and medio-dorsal thalamus) and vermis cerebelli. An investigator blinded for clinical data classified the pathological changes semiquantitatively for each section (0–4 points for no change, mild, moderate, severe or maximal changes) [21,22]. The intensity was estimated for spongiform changes and nerve cell loss. For gliosis, the quantity of glial proliferation, including nuclear pleomorphy and gemistocytic changes of the cytoplasm, was assessed. A status spongiosus with collapsing tisssue matrix, severe astrocytic gliosis and nearly complete nerve cell loss was considered as the maximal change. The reliability of the neuropathological scoring profiles was assessed before and revealed comparable results between the investigators in former investigations [22].

### Biochemical CSF analysis

The routine investigations of the CSF did not reveal any abnormalities with respect to cell count and protein profile.

All CSF samples were analyzed with respect to 14-3-3 proteins, neuron specific enolase (NSE), tau, phosphorylated tau, S-100b, PrP^c ^and Aβ_1–42 _in the reference laboratory of the Surveillance Unit in Göttingen according to standards described previously [2,23-26]

The 14-3-3 capture kit, Repairgenics, Bioproducts (Mainz, Germany, until July 2003, now Schwerin, Germany), was used for 14-3-3 detection, LIAISON^® ^NSE for NSE detection, LIAISON SANGTEC^® ^100 for S-100b detection, INNOTEST™ hTauAg by INNOGENETICS for tau detection, INNOTEST™ Phospho Tau for the detection of the phosphorylated tau at residue 181, INNOTEST™ β-Amyloid (1–42) for Aβ-Amyloid. PrP^c ^(comprising PrP^C ^and potentially to minor degree PrP^Sc^) concentrations were determined by using a commercially available ELISA for detection of the abnormal PrP^Sc ^(Platelia BSE detecton kit, BIO-RAD Laboratories GmbH, Munich, Germany). The following modifications were made to the test protocol: the proteinase K digestion step, which is used to degrade the normal, but not the abnormal form of the prion protein, was omitted, since we were interested in detecting all PrP^c ^which was present in the sample. To quantify levels of PrP, a standard curve using recombinant human prion protein (Prionics AG, Zurich, Switzerland) was used in each experiment, according to another PrP-ELISA protocol [25,27].

NSE was determined in all 57 patients, tau protein in 56, phosphorylated tau in 33, S-100b protein in 54, Aβ_1–42 _in 41, PrP^c ^in 45 and 14-3-3 in 51 cases.

### Statistical analysis

A regression analysis with respect to the degree of neuropathological changes in all nine examined brain regions and the concentrations of the CSF markers was performed.

The statistics software package we used for our calculations was SigmaStat 3.1 and SigmaPlot 9.0 by Systat Software Inc., Point Richmond, USA. A linear regression and the calculation of the Pearson Product Moment Correlation Coefficient were performed for the analysis of any trends between neuropathological lesion profiles and concentrations of CSF markers.

## Results

### Study population

Clinical data on 57 sCJD cases and the CSF concentration of tau, phosphorylated tau, 14-3-3, S100b, NSE, PrP^c ^and Aβ_1–42 _are given in Tables 1 and 2. Looking at the time of the lumbar puncture within the whole disease course, 6 patients had their CSF taken in the first third, 12 patients in the second third and 39 patients during the last third of the disease course (Table 1). The stratification of the data by time of lumbar puncture and the exclusion of single cases with extremely long disease duration or a lumbar puncture very early at onset did not change the results presented here (data not shown).

### Correlation of neuropathological lesion profiles and concentrations of brain-derived proteins in the CSF

The degree of neuropathological changes (spongiform changes, neuronal loss and gliosis) in nine defined brain areas was analyzed. The severity of spongiform changes correlates with neuronal loss and gliosis (data not shown).

A regression analysis of the neuropathological lesion profiles and the concentrations of the CSF markers was carried out. The correlation coefficients were calculated and significant results (p ≤ 0.05) are indicated (Tables 3, 4, 5).

We analyzed if there is a correlation between the NSE concentration in the CSF and neuropathological changes. The NSE concentrations showed no correlation with the degree of spongiform change, gliosis and nerve cell loss in the cortical regions. However, elevated NSE levels correlated significantly with the degree of gliosis in the basal ganglia and cerebellum and with the degree of spongiform changes in the thalamus.

The tau protein levels correlated negatively with the degree of neuropathological changes in cortical regions. A positive correlation was also found between the degree of spongiform changes and gliosis in the cerebellum. The tau concentrations showed no correlation with the degree of spongiform changes and neuronal loss in the basal ganglia, in contrast to the degree of gliosis in the basal ganglia, where a positive correlation was found.

The levels of phosphorylated tau concentrations show negative correlations with the degree of neuropathological changes in cortical regions. A negative correlation was also found between the degree of neuronal loss and gliosis in the thalamic region, whereas a positive correlation was found between spongiform changes and the thalamus. Spongiform changes in the cerebellum correlated in a positive way with phosphorylated tau levels. No correlation was found between the degree of neuropathological changes and phosphorylated tau levels.

14-3-3 protein levels correlated negatively with the degree of spongiform changes in the cortical regions and with the degree of neuronal loss in the thalamus. The 14-3-3 protein levels showed a slightly positive correlation with the degree of spongiosis in the thalamus. For other brain regions, no correlation was observed.

The S 100b concentrations correlated negatively with neuropathological changes cortical regions and with the degree of gliosis and nerve cell loss in the thalamus. In contrast to this, a positive correlation was found with the degree of spongiform change in the thalamus and the degree of gliosis in the basal ganglia and cerebellum.

PrP concentrations in the CSF were negatively correlated with the degree of neuropathological changes in most cortical regions. A negative correlation was also found with the degree of gliosis and neuronal loss in the basal ganglia. The PrP levels correlated negatively with the degree of neuronal loss in the cerebellum and the degree of gliosis in the thalamus. However, PrP^c ^levels correlated significantly in a positive way with the degree of spongiform changes in the cerebellum.

The Aβ_1–42 _concentrations were negatively correlated with the degree of neuropathological changes in all cortical regions and the basal ganglia. A negative correlation was also found between the degree of neuronal loss and gliosis in the thalamus. Aβ1–42 concentrations correlated positively with spongiform changes in the cerebellum.

A synopsis on correlation of various brain-derived proteins and degree of pathological changes in various cortical and subcortical regions is shown in Table 6. In general, for cortical lesions, NSE levels do not correlate with severity of pathological changes (they do not increase further with lesion severity), whereas all other proteins analyzed showed a tendency towards lower levels with the increasing severity of pathological changes. This effect was more pronounced for Aβ_1–42 _and neuronal loss in temporal regions, cingulated gyrus and parietal regions. The analysis of the pathological changes in subcortical areas showed more diverse results. The degree of pathological changes in the basal ganglia correlated with a tendency of NSE levels to increase and PrP^c ^and Aβ_1–42 _levels to decrease. The severity of neuronal loss and gliosis in the thalamus correlated negatively with concentrations of Aβ_1–42_, S-100b and phosphorylated tau levels. A positive correlation was seen for spongiform changes and levels of 14-3-3, NSE, S 100b, and phosphorylated tau.

**Figure 1:**
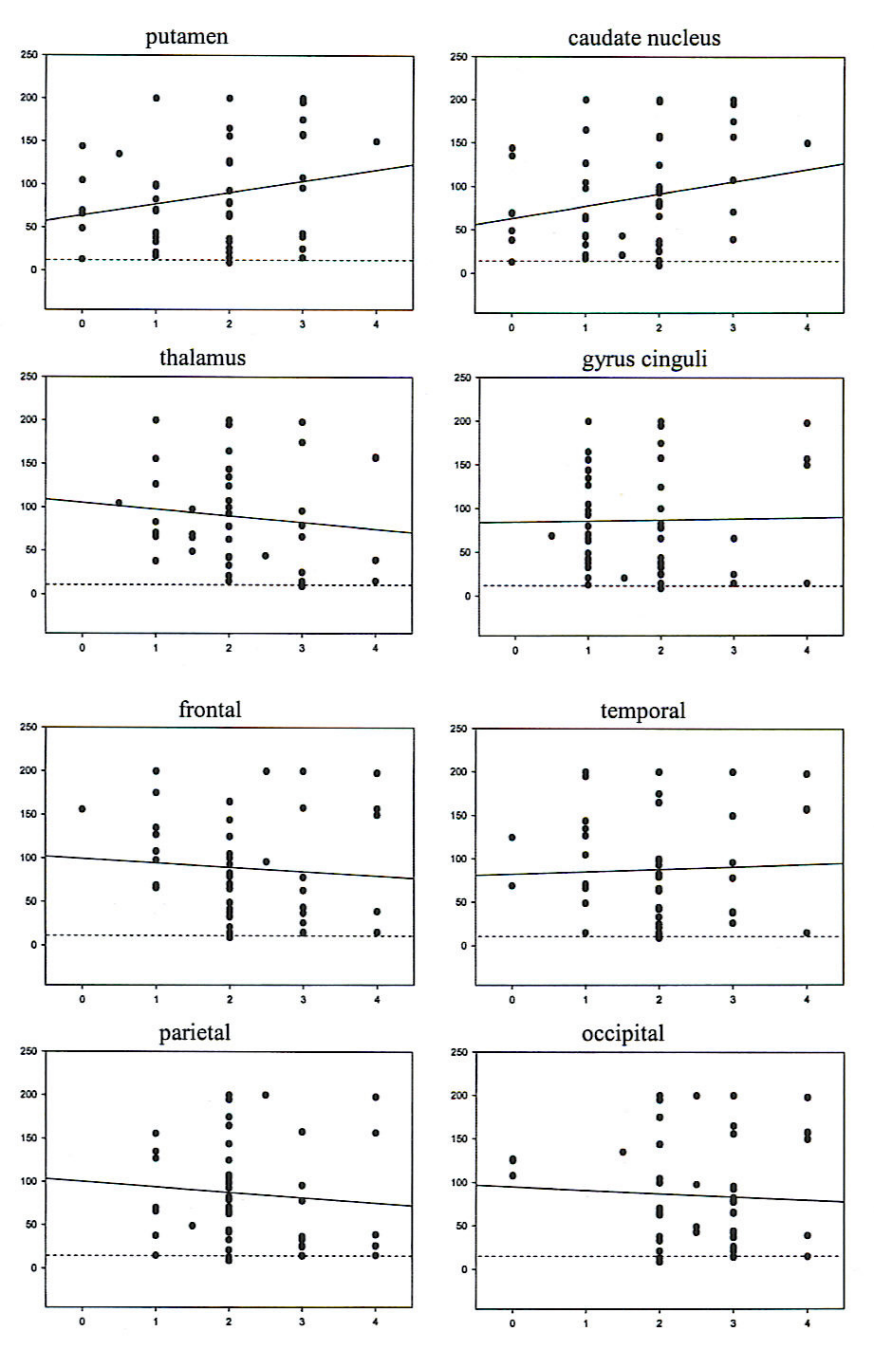
Correlation between CSF levels of neuron-specific enolase and degree of neuronal loss in various brain areas. (- - - - - - cut-off 12.5 ng/ml).

**Figure 2:**
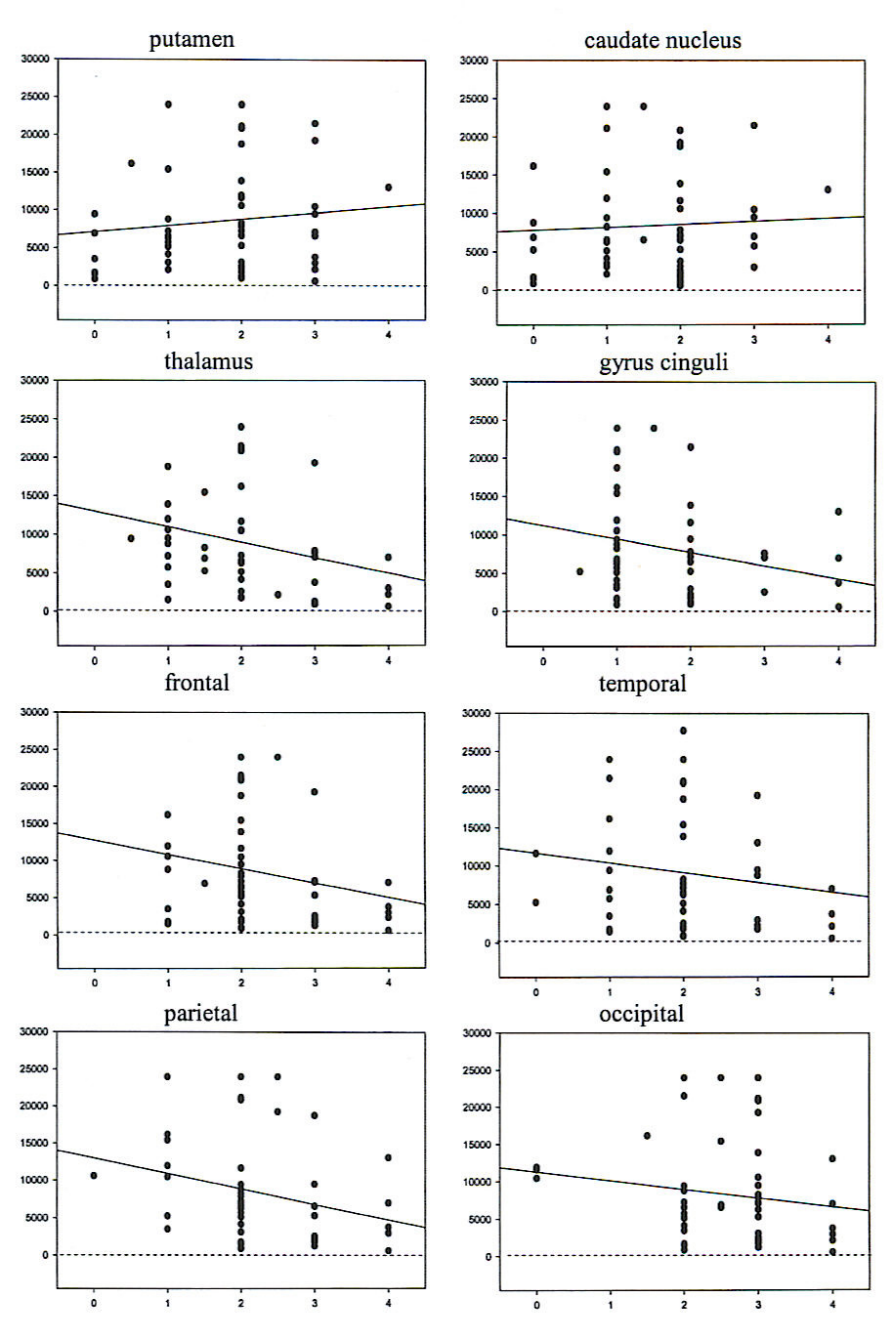
Correlation between tau CSF levels and degree of neuronal loss in various brain areas. (- - - - - - cut-off 195 pg/ml).

**Figure 3:**
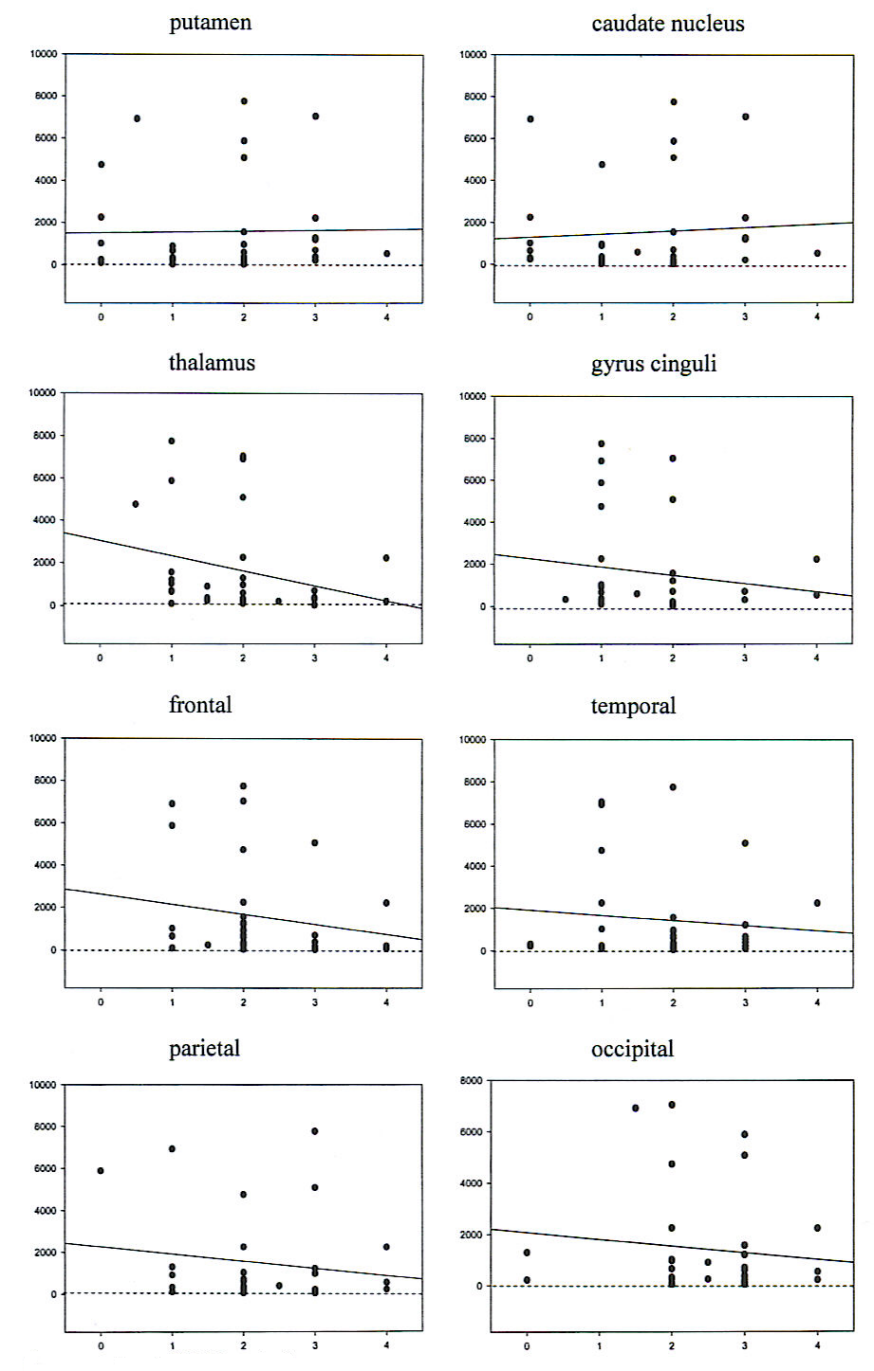
Correlation between 14-3-3 CSF levels and degree of neuronal loss in various brain areas. (- - - - - - cut-off 200 ng/ml).

**Figure 4:**
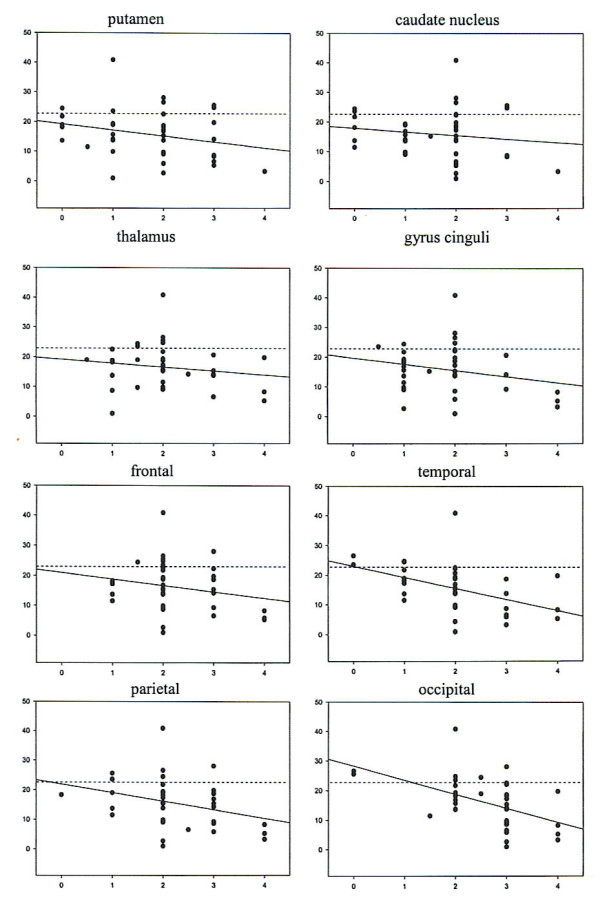
Correlation between PrP CSF levels and degree of neuronal loss in various brain areas. (- - - - - - cut-off 22 ng/100 ml).

**Figure 5:**
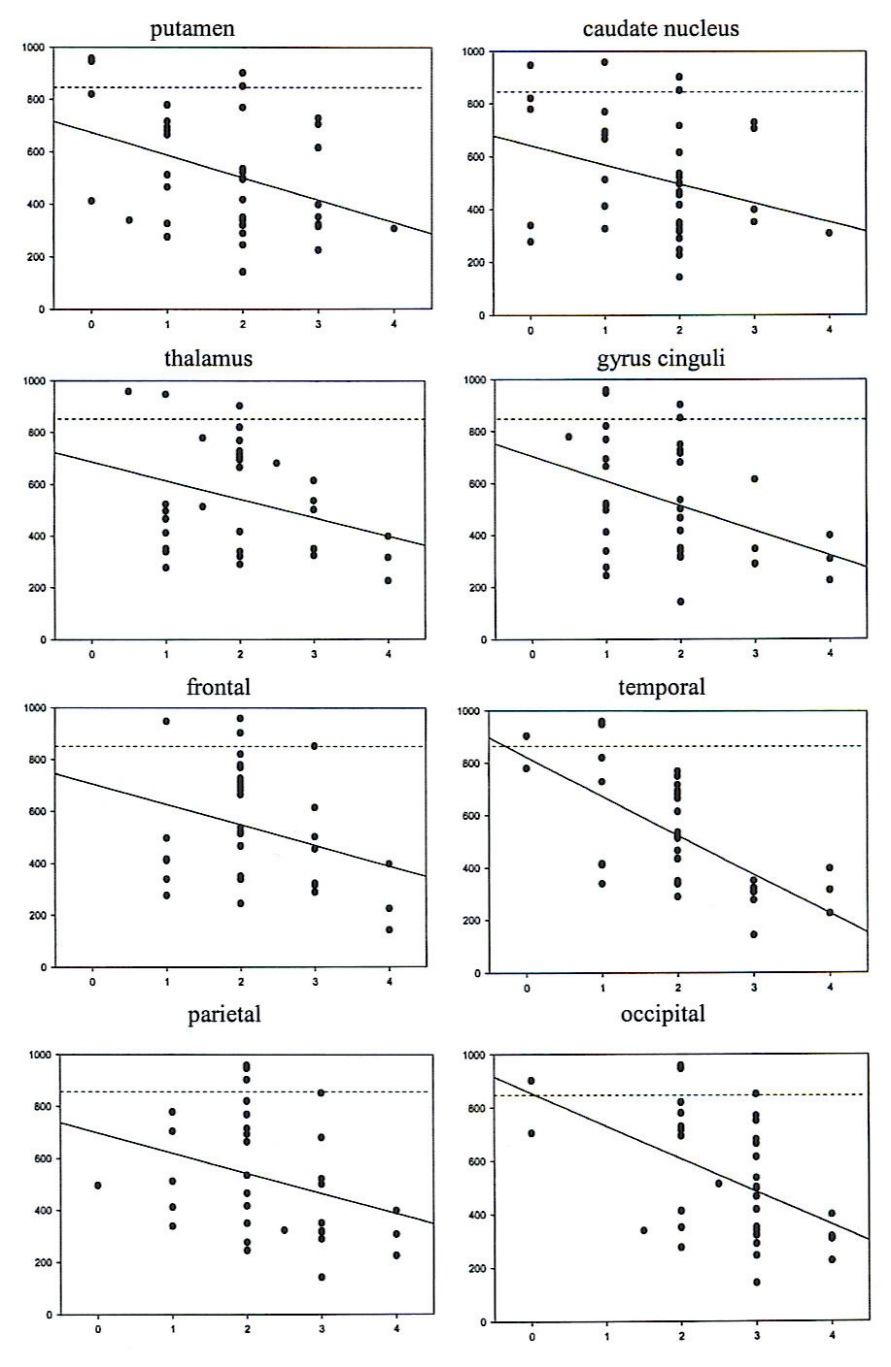
Correlation between Aβ_1–42 _CSF levels and degree of neuronal loss in various brain areas. (- - - - - - cut-off 849 pg/ml).

Figures 1, 2, 3, 4, 5 show the correlation between degree of neuronal loss in various brain areas and CSF levels of NSE, tau, 14-3-3, Aβ_1–42 _and PrP^c^.

## Discussion

In CJD patients, most attention has been concentrated on investigating the role and the biochemical properties of the pathological form of the prion protein (PrP^Sc^) in central nervous system tissue. Brain derived proteins were mainly studied with respect to their clinical diagnostic potential rather than to reflect the severity of brain lesions in CJD. The mechanisms of elevation of brain-derived proteins in the CSF in patients with CJD and other acute neurological diseases are not known in detail and the current (yet unproven) hypothesis suggests a leakage into the CSF following rapid neuronal damage [1,2,28-30].

The earliest markers studied in CJD were NSE and S 100b proteins, which were shown to be elevated in the CSF during the disease progression, but a subsequent study was done only in one patient with repeated lumbar punctures [13]. In other neurological diseases, high NSE levels in CSF and serum were used as a prognostic marker of acute neuronal damage (e.g. hypoxia or ischemia) and disease progression [31,32]. In CJD, NSE concentrations increased to a maximum when the disease activity was most prominent and returned to normal or mildly elevated levels in the terminal stage [13]. These results imply that these protein levels can serve as biochemical markers for the presence of an acute neuronal loss in CJD brain [13]. In addition, other studies assumed that measurement of the NSE might correlate with the disease progression and NSE levels decrease with reduced numbers of remaining neuronal cells [15].

We observed that NSE levels increase with severity of neuronal lesions already when only minor changes take place and they are clearly correlated to the damage of subcortical grey matter nuclei, in particular the thalamus, but also the basal ganglia. Of interest, NSE levels increase when the degree of cortical and subcortical changes is small and do not increase further with more pronounced neuronal damage or gliosis of cortical structures. NSE was the only marker for which we observed such a correlation.

Elevated levels of protein 14-3-3 were reported in 80–95% of patients with sCJD [1,2,18]. The amount of 14-3-3 in the CSF is thought to be linked to the degree of neuronal destruction and to the disease stage. In our study, 14-3-3 levels correlated negatively with the degree of spongiform changes in cortical areas and with the degree of neuronal loss in the thalamus.

In our study, the highest correlation coefficients were obtained for Aβ_1–42 _concentrations and the degree of spongiform change, gliosis and nerve cell loss in the inferior temporal gyrus, the area striata and cingulate gyrus. Significant correlation was found for the degree of spongiform changes in the cerebellum and Aβ_1–42_. There were also high correlations between phosphorylated tau concentrations and the degree of gliosis in most cortical regions, the degree of nerve cell loss in the area striata and the degree of spongiform change and nerve cell loss in the inferior parietal gyrus.

Tau and Aβ_1–42 _levels were initially studied in patients with Alzheimer's disease and only later were shown to follow a similar pattern in CJD (elevated tau and decreased Aβ_1–42 _levels). The pathogenesis of tau-elevation in the CSF in various forms of dementia is thought to be attributable to the degree of neuronal cell death [33], but an early increase of CSF tau in AD is not explained by this hypothesis [34]. A recent study demonstrated that the CSF tau level correlates significantly with right frontal and left temporal cortical atrophy in Alzheimer's disease [35]. These results are partly in line with our observations in CJD on a significant correlation between tau levels and the degree of spongiform changes in the frontal cortex.

Tau protein levels in the CSF have been studied with respect to disease duration and disease stage in CJD [12,36]. Tau concentrations were lower in CJD patients with a long duration of disease and were lowest at the onset or at the end stage of the disease [12]. Our data explain and extend this observation. After an increase with minor lesion severity, tau protein levels decrease, but stay abnormal in the CSF with more severe cortical lesions in CJD.

The PrP^c ^concentrations showed a high correlation with the degree of gliosis and nerve cell loss in the area striata and the inferior temporal gyrus and with spongiform changes in the cerebellum. Levels of PrP^c ^and Aβ_1–42 _clearly correlated with the degree of pathology in cortical structures, but not with basal ganglia and thalamic pathology in CJD. This pattern was consistent in the analysis of various cortical regions and the most striking effect was seen for Aβ_1–42 _when compared to the degree of lesion severity in the temporal lobe and cingulate gyrus.

## Conclusion

Taken together, we observed three different patterns of CSF protein levels associated with the degree of cortical and subcortical changes. The first one was seen for NSE. Levels increased with only mild cortical and subcortical changes and increased further with the degree of subcortical changes.

The second pattern was seen for tau and 14-3-3. There is a clear elevation of the levels of these proteins in the CSF in disease stages when the degree of spongiform changes, neuronal loss and gliosis in cortical areas are relatively small. In later stages, the levels correlate with the degree of neuronal damage in cortical structures and decline. One can only speculate on the mechanisms for this finding; a potential for increased synthesis or upregulation at early stages when the brain damage is relatively small cannot completely be excluded [37,38].

A completely different mechanism must be discussed for PrP^c ^and Aβ_1–42_. Normal levels are measured in the CSF for those proteins at initial stages. With increasing severity of cortical lesions they decrease. Levels of PrP^c ^and Aβ_1–42 _levels are independent of the degree of the brain damage in subcortical areas. Since PrP^c ^is mainly synthesized in neurons, one can assume that PrP^c ^CSF levels are determined by the degree of neuronal loss, mainly in cortical areas [39]. Among the latter, pathological changes in the inferior temporal lobe seem to have the most effect on PrP^c ^and Aβ_1–42 _levels in the CSF.

To conclude, brain specific lesion patterns have to be considered when analyzing CSF neuronal proteins.

## Competing interests

The author(s) declare that they have no competing interests.

## Authors' contributions

CB-G, WJS-S and IZ conceived of the study concept and design. CB-G, WJS-S, MB, AK, BM, UH, DV and AG were responsible for acquisition of data. CB-G, AK, UH, BM and IZ carried out the analysis and interpretation of data. CB-G, WJS-S and IZ participated in the drafting of the manuscript. IZ analysed and revised the manuscript for important intellectual content. HAK and IZ obtained funding. MB, BC and SE provided administrative, technical and material support. IZ supervised the study.

All authors read and approved the final manuscript.

## Pre-publication history

The pre-publication history for this paper can be accessed here:



## Figures and Tables

**Table 1:** Clinical characteristics of the patients included in our study (n = 57)

Sex	27 males, 30 females; male-female ratio 0.9
Age at disease onset (median)	61.5 (23–81)
Codon 129 genotype (n = 55)	MM: 39 MV: 3 VV: 13 n.d.: 2
PrP^Sc^	type 1: 32 type 2: 9
Duration of disease (months)	4.26 (1.1–34.3)
Onset until lumbar punction (months)	3.02 (0.3–27.8)
Lumbar punction until death (months)	0.94 (0–30.8)

**Table 2:** Concentrations of the cerebrospinal fluid (CSF) markers in CJD

**CSF marker**	**median (range)**	reference cut-off*
NSE (n = 57)	70 (9–200) ng/ml	12.5 ng/ml
tau (n = 56)	6764 (3772–27770) pg/ml	195 pg/ml
phosphorylated tau (n = 33)	55 (18.4–138) pg/ml	61 pg/ml
14-3-3 (n = 51)	984 (410–5387) ng/ml	200 ng/ml
S 100b (n = 54)	8 (1.4–39.2) ng/ml	2 ng/ml
PrP (n = 45)	15.7 (1–40.9) ng/100ml	22 ng/100ml
Aβ_1–42_	467 (143–959) pg/ml	849 pg/ml

*: data were taken according to the manufacturers instruction in cases where commercially developed kits are available, otherwise calculated based on results from non-neurological controls from our study (14-3-3 and PrP)

**Table 3:** Severity of lesion and concentrations of CSF markers (correlation coefficient): Spongiform changes

	**Cortical regions**					**Subcortical regions**			**Cerebellum**
	**CG°**	**MFG°**	**ITG°**	**IPG°**	**AS°**	**Putamen**	**Caudate ncl.**	**MDT°**	

**NSE**	0.0709	0.0731	-0.0351	-0.0485	-0.0267	0.164	0.14	0.305*	0.034
**Tau**	-0.173	-0.313*	-0.219	-0.309*	-0.217	-0.0158	-0.0963	0.024	0.239
**Tau phosphorylated**	-0.302	-0.0729	-0.227	-0.440*	-0.195	-0.0431	0.0466	0.282	0.375
**14-3-3**	-0.142	-0.0968	-0.275	-0.317	-0.198	0.0631	0.190	0.246	0.179
**S 100b**	-0.0686	-0.0459	-0.303*	-0.282	-0.359*	0.0893	0.0864	0.242	-0.005
**PrP**	-0.276	-0.0955	-0.307	-0.364*	-0.335*	0.0109	-0.0289	0.00314	0.451*
**Aβ**_**1–42**_	-0.453*	-0.233	-0.560*	-0.356*	-0.426*	-0.159	-0.248	0.0489	0.465*

° MDT: medio-dorsal thalamus

CG: cingulate gyrus

MFG: medial frontal gyrus

ITG: inferior temporal gyrus

IPG: inferior parietal gyrus

AS: area striata

* significant, p ≤ 0.05

**Table 4:** Severity of lesion and concentrations of CSF markers (correlation coefficient): Neuronal loss

	**Cortical regions**					**Subcortical regions**			**Cerebellum**
	**CG°**	**MFG°**	**ITG°**	**IPG°**	**AS°**	**Putamen**	**Caudate ncl.**	**MDT°**	

**NSE**	0.0183	-0.0846	0.0428	-0.0769	-0.0586	0.216	0.216	-0.114	0.202
**Tau**	-0.233	-0.229	-0.159	-0.276	-0.158	0.121	0.0516	-0.257	-0.140
**Tau phosphorylated**	-0.216	-0.236	-0.337	-0.454*	-0.505*	-0.128	-0.130	-0.235	-0.058
**14-3-3**	-0.146	-0.170	-0.103	-0.154	-0.118	0.0184	0.0698	-0.266	-0.127
**S 100b**	-0.169	-0.203	-0.192	-0.344*	-0.282*	0.195	0.199	-0.254	-0.0115
**PrP**	-0.240	-0.219	-0.457*	-0.318	-0.511*	-0.260	-0.144	-0.159	-0.279
**Aβ**_**1–42**_	-0.386	-0.295	-0.661*	-0.315	-0.488*	-0.387	-0.302	-0.321	-0.0136

° MDT: medio-dorsal thalamus

CG: cingulate gyrus

MFG: medial frontal gyrus

ITG: inferior temporal gyrus

IPG: inferior parietal gyrus

AS: area striata

* significant, p ≤ 0.05

**Table 5:** Severity of lesion and concentrations of CSF markers (correlation coefficient): Gliosis

	**Cortical regions**					**Subcortical regions**			**Cerebellum**
	**CG°**	**MFG°**	**ITG°**	**IPG°**	**AS°**	**Putamen**	**Caudate ncl.**	**MDT°**	

**NSE**	-0.0553	-0.0280	0.0104	-0.0345	-0.0445	0.298*	0.265*	0.0413	0.386*
**Tau**	-0.242	-0.174	-0.178	-0.160	-0.207	0.141	0.254	-0.0726	0.236
**Tau phosphorylated**	-0.423*	-0.175	-0.496*	-0.479*	-0.536*	-0.131	-0.111	-0.260	-0.090
**14-3-3**	-0.189	-0.155	-0.185	-0.0348	-0.114	0.0775	0.198	-0.132	-0.110
**S 100b**	-0.261	-0.188	-0.195	-0.271	-0.245	0.262	0.323*	-0.236	0.321
**PrP**	-0.344*	-0.260	-0.426*	-0.383*	-0.511*	-0.250	-0.168	-0.409*	-0.026
**Aβ**_**1–42**_	-0.517*	-0.387*	-0.495*	-0.396*	-0.462*	-0.306	-0.357*	-0.387*	0.038

° MDT: medio-dorsal thalamus

CG: cingulate gyrus

MFG: medial frontal gyrus

ITG: inferior temporal gyrus

IPG: inferior parietal gyrus

AS: area striata

* significant, p ≤ 0.05.

**Table 6:** Synopsis of the degree of pathological changes and levels of various brain-derived proteins in the CSF

	Spongiform change/neuronal loss/gliosis *			
	
	**Cortical Regions**	**Subcortical Regions**		**Cerebellum**
			
		**basal ganglia**	**thalamus**	
**NSE**	↔/↔/↔	↔/↑/↑	↑/↔/↔	↔/↑/↑
**tau**	↓/↓/↔	↔/↔/↑	↔/↓/↔	↑/↔/↑
**phosphorylated tau**	↓/↓/↓	↔/↔/↔	↑/↓/↓	↑/↔/↔
**14-3-3**	↓/↔/↔	↔/↔/↔	↑/↓/↔	↔/↔/↔
**S 100b**	↓/↓/↓	↔/↔/↑	↑/↓/↓	↔/↔/↑
**PrP**	↓/↓/↓	↔/↓/↓	↔/↔/↓	↑/↓/↔
**Aβ**_**1–42**_	↓/↓**/↓	↓/↓/↓	↔/↓/↓	↑/↔/↔

*: within the cells in the table the first arrow shows spongiform change, the second shows nerve cell loss and the third gliosis; only the strongest neuropathological changes among the different regions are shown in the table

**: strong correlation

↔ correlation coefficient from -0.2 to +0.2: no correlation

↑ correlation coefficient from +0.2 to +0.6: weak correlation

↓ correlation coefficient from -0.2 to -0.6: weak correlation.
